# Peculiarities of the Interaction of the Bacteriolytic Protease Blp from *Lysobacter capsici* XL1 with the Cell Wall of *Staphylococcus aureus* 209P

**DOI:** 10.3390/ijms27125246

**Published:** 2026-06-10

**Authors:** Irina Kudryakova, Alexey Afoshin, Egor Bulavko, Dmitry Ivankov, Bogdan Melnik, Elena Leontyevskaya, Natalia Leontyevskaya

**Affiliations:** 1 Laboratory of Microbial Cell Surface Biochemistry, G.K. Skryabin Institute of Biochemistry and Physiology of Microorganisms, FRC PSCBR, Russian Academy of Sciences, 5 Prosp. Nauki, 142290 Pushchino, Russia; kudryakovairina@yandex.ru (I.K.); alex080686@mail.ru (A.A.); ealeont@gmail.com (E.L.); 2 Center for Bio- and Medical Technologies, 121205 Moscow, Russia; e.bulavko@ligandpro.ru (E.B.); ivankov13@gmail.com (D.I.); 3 Institute of Protein Research, Russian Academy of Sciences, 4 Institutskaya Str., 142290 Pushchino, Russia; bmelnik@phys.protres.ru

**Keywords:** *Lysobacter*, bacteriolytic protease Blp, C-terminal subdomain, bacteriolytic protease–target cell interaction, *Staphylococcus aureus* 209P peptidoglycan, site-directed mutagenesis

## Abstract

The *Lysobacter capsici* XL1 β-lytic protease (Blp) is a bacteriolytic enzyme that hydrolyzes peptide bonds in the interpeptide bridge of the peptidoglycan of Gram-positive bacteria, including antibiotic-resistant strains of pathogenic bacteria. The Blp has been extensively characterized. The only unexplored aspect is the mechanism by which this enzyme recognizes target cells. In this work, we demonstrated for the first time that the Blp structure contained a C-terminal subdomain that can be responsible for this interaction. Molecular modeling suggested a hydrophobic nature of the interaction between the Blp and peptidoglycan. Model mutant forms of the Blp, which have fewer hydrophobic areas in the C-terminal subdomain, also had fewer sites for potential interaction with the ligand. Wet lab experiments showed that these mutant Blp forms exhibited poorer binding to peptidoglycan and living *Staphylococcus aureus* 209P cells, resulting in decreased bacteriolytic and proteolytic activity. Amino acid residues N136 and Y160 in the C-terminal subdomain were identified and can be important for the interaction of the enzyme with target cells. Further research into the mechanism of target cell recognition by bacterial bacteriolytic proteases will enable the use of this knowledge to expand the specificity of action of these enzymes, including as antimicrobial agents for medical applications.

## 1. Introduction

Extracellular bacteriolytic proteases are a group of secreted enzymes that exhibit specific activities towards the peptidoglycan of target bacterial cells. Peptidoglycan consists of linear glycan chains cross-linked by short peptides. Glycan chains are composed of alternating residues of N-acetylglucosamine (NAG) and N-acetylmuramic acid (NAM) linked by β-1,4 glycosidic bonds. A peptide chain (peptide stem) is attached to the N-acetylmuramic acid residue. Peptide chains are connected to each other by a peptide bond either directly, as in Gram-negative bacteria, or via a short peptide bridge, as in most Gram-positive bacteria. In peptidoglycan, bacteriolytic proteases hydrolyze peptide bonds in the peptide stem and/or in the interpeptide bridge, if present, leading to the death of the target cell. These enzymes also hydrolyze peptide bonds in proteins. The mechanisms of peptide bond catalysis by these enzymes are well studied [1,2]. However, the question of the initial stage of interaction of bacteriolytic proteases with the surface of target cells—whether there is a specific mechanism for recognizing the target cell—remains open.

The β-lytic protease (EC 3.4.24.32), according to the MEROPS database [3], is a Zn-dependent metalloproteinase of the M23A family. The protease was first isolated in 1965 from the culture fluid of the Gram-negative bacterium *Lysobacter enzymogenes* M497-1 (formerly *Sorangium* sp.) [4]. We have described the β-lytic protease (Blp) of *L. capsici* VKM B-2533^T^ relatively recently [5]. Today, it can be considered one of the best-studied extracellular bacteriolytic proteases in Gram-negative bacteria. The enzyme is biochemically characterized; hydrolysis sites in protein substrates, including previously unknown ones, are detected; the spatial structure of the protein is solved; antimicrobial activity against living *Staphylococcus* cells, including MRSA strains, *Micrococcus luteus* Ac-2230^T^, *Kocuria rosea* Ac-2200^T^, *Enterococcus faecium* FS86, *Curtobacterium flaccumfaciens* pv. *flaccumfaciens*, is established. A homologous expression system for the Blp has been developed, which allows the enzyme to be produced in biotechnologically significant amounts and, most importantly, without loss of lytic activity [5,6,7,8]. Among the known bacterial extracellular proteases of the M23 family, namely lysostaphin, pseudoalterin, staphylolysin, zoocin A, enterolysin A, ALE-1, SpM23_A, and SpM23_B [9], the Blp enzyme from *L. capsici* has the broadest spectrum of antibacterial and proteolytic activity.

However, the mechanism of Blp interaction with target cells remains an open question. The problem has not been solved for other known extracellular bacteriolytic proteases of Gram-negative bacteria, either. The fact is that these enzymes feature a single-domain organization—unlike the extracellular enzymes of Gram-positive bacteria, which are characterized by a two-domain organization and the presence of a substrate-binding domain. For example, in lysostaphin from the Gram-positive bacterium *S. simulans* biovar *staphylococcus*, this type of enzyme organization has been very well studied [10,11]. The only study of the binding mechanism of the extracellular bacteriolytic protease pseudoalterin from the Gram-negative bacterium *Pseudoalteromonas* sp. CF6-2 has been published fairly recently [12]. The authors identified a region in the protein’s spatial structure responsible for binding the carbohydrate moiety of bacterial peptidoglycan. We hypothesized that the Blp structure might also contain such a region, a subdomain, responsible for the enzyme’s initial interaction with the surface of target cells. The identification of such a subdomain in the Blp enzyme was the primary goal of this work.

## 2. Results

### 2.1. Identification of a Possible Binding Site for Bacterial Peptidoglycan in the Structure of the Bacteriolytic Protease Blp

**Figure 1:**
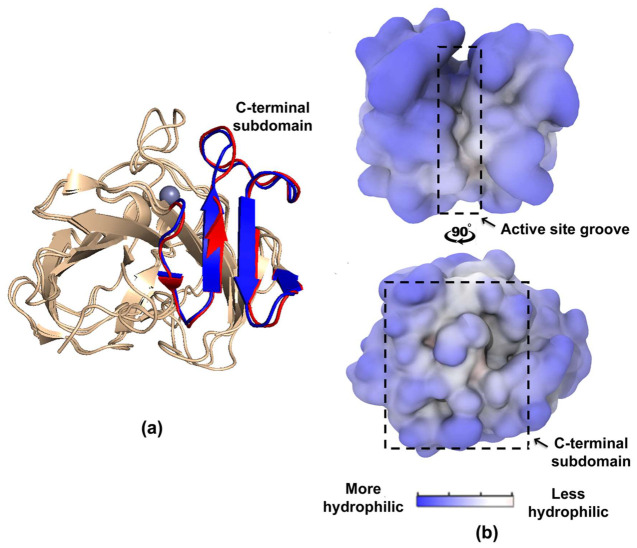
Identification of a potential bacterial peptide-binding site in the structure of the bacteriolytic protease Blp. (**a**) Structural superposition of the bacteriolytic protease Blp with pseudoalterin. The C-terminal subdomain of the Blp and pseudoalterin proteases are shown in blue and red, respectively. (**b**) Surface hydrophobicity/hydrophilicity map of the bacteriolytic protease Blp.

The bacteriolytic proteases Blp and pseudoalterin belong to the same family of metalloproteases, M23A. We performed a structural superposition of these enzymes and established their homology (Figure 1a).

The Blp structure revealed a segment at amino acid residues N136–R167, consisting of four antiparallel β-strands, which is homologous to the C-terminal subdomain of amino acid residues N134–R173 of pseudoalterin. It is known that this particular segment in the pseudoalterin structure is responsible for binding to the carbohydrate moiety of bacterial peptidoglycan [12]. We hypothesized that the segment at amino acid residues N136–R167 in the Blp structure may perform a similar function.

To understand the nature of possible interactions between the Blp and its substrate, we constructed a hydrophobicity/hydrophilicity map of the protein surface combined with molecular dynamics (MD) simulations of the enzyme with its ligand. A fragment of the carbohydrate backbone of peptidoglycan N-acetylglucosamine–N-acetylmuramic acid (NAG–NAM)_2_ was used as a ligand. Two regions of lower hydrophilicity were identified: in the catalytic groove and the C-terminal subdomain of the enzyme (Figure 1b). MD simulations of the enzyme with the (NAG–NAM)_2_ ligand showed that the ligand interacted with the C-terminal subdomain of the enzyme. However, we could not identify a single or a number of distinct structurally stable conformations for the ligand attached to the protein. Instead, (NAG–NAM)_2_ showed substantial flexibility of its carbohydrate backbone and most of side chain moieties. The bound state can be characterized solely by one or several of the acetamide groups of the ligand interacting with the pocket-like regions of the C-terminal subdomain with lower hydrophilicity, which are formed by amino acid residues N136, Y139, R144, T146, T148, Y160, T162, N164 (Figure S1). Within such regions, no remarkable hydrogen bond or salt bridge networks between ligand and nearby protein residues were observed, which is indicative of a predominantly hydrophobic nature of interaction between the bacteriolytic protease Blp and the (NAG–NAM)_2_ ligand. The average lifetime of this kind of interactions was determined to be about 10–50 ns of MD simulation (the free energy of binding of one acetamide group of ligand to protein is estimated to be approximately 1–5 kcal/mol according to transition state theory), and they could be rapidly disrupted by increasing the temperature to 350 K or higher. It is also worth noting that the lactyl group of NAM in the observed complexes is exposed to solvent and barely interacts with the protein (Figure S1).

**Figure 2:**
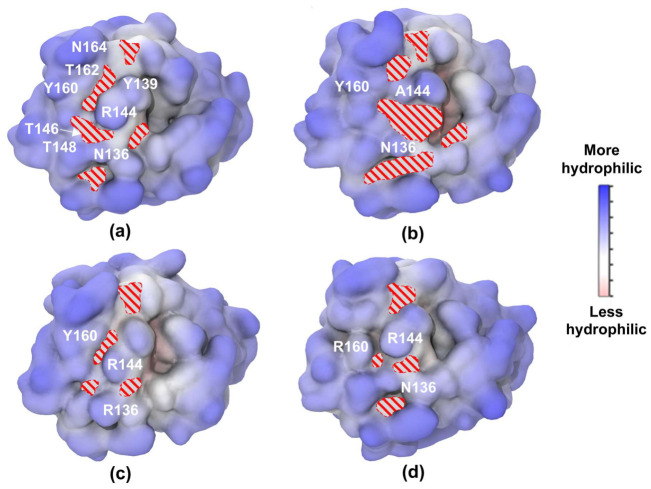
Surface hydrophobicity/hydrophilicity map of the bacteriolytic protease Blp (**a**) and model mutant proteins R144A (**b**)**,** N136R (**c**)**,** and Y160R (**d**). Red dashes indicate the locations of the acetamide groups of the (NAG–NAM)_2_ ligand in the pocket-like regions of the C-terminal subdomain of the proteins.

Residues Y160, R144, and N136, located at the interface of several segments with lower hydrophilicity, were chosen to study the protein–ligand interaction. This particular choice was motivated by insights of C-terminal subdomain surface organization obtained from MD simulations of wild-type Blp (Figure 2a). R144 and Y160 (along with Y139 and T162) border a highly occupied by acetamide groups pocket located in the central part of the subdomain. In turn, N136 separates two more zones, which together with the central zone interact with 75% of the acetamide groups of the ligand within the putative binding site. To confirm the role of these residues in the interaction with the (NAG–NAM)_2_ ligand, we performed an MD simulation to determine the effect of point substitutions of the selected residues on the hydrophobicity/hydrophilicity profile of the Blp and its affinity for the ligand. Mutant proteins with substitutions of tyrosine at position 160 with alanine (Y160A) and arginine (Y160R), arginine at position 144 with alanine (R144A), and asparagine at position 136 with alanine (N136A) and arginine (N136R) were modeled. The choice of amino acid substitutions was determined by their possible impact on the physicochemical parameters of the surface of the bacteriolytic protease Blp. As a result, changes in the hydrophobicity/hydrophilicity profile of the C-terminal subdomain of the protein and in the protein–ligand interaction were revealed in the model mutant proteins R144A, N136R, and Y160R. Thus, for the R144A protein, the hydrophobic surface area in the region of the putative binding site increased by 29% compared to the native form of Blp (Figure 2b), whereas for the N136R and Y160R proteins, it decreased by 17% and 14%, respectively (Figure 2c,d). No obvious changes in the hydrophobicity profile of the proteins N136A and Y160A were detected compared to the wild-type Blp protease. From the mechanistic point of view, the substitution of R144 by a potentially less sterically obscuring and hydrophobic alanine resulted in the spatial expansion of one of the nearby pockets. Mutation N136R, on the contrary, made the corresponding pocket unreachable due to its occupation by arginine side chain. Finally, Y160 replaced by arginine resulted in a decrease in the effective size of the central pocket by the latter.

Thus, it can be hypothesized that the substitution of arginine with alanine at position 144 increases the binding affinity of the Blp protease to peptidoglycan, while the substitution of asparagine at position 136 and tyrosine at position 160 with arginine decreases it. This hypothesis was further confirmed experimentally.

### 2.2. Production of Mutant Forms of the Blp Bacteriolytic Protease

**Figure 3:**
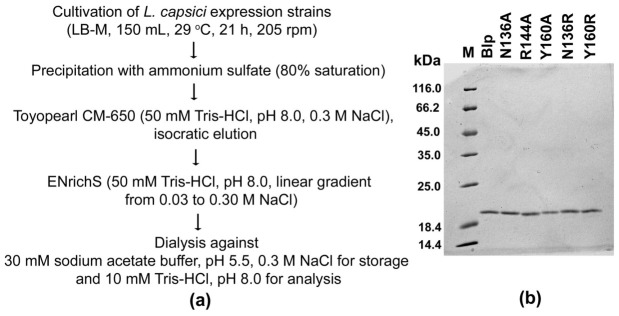
Preparation of the bacteriolytic protease Blp and its mutant forms. Scheme of enzyme purification (**a**). SDS-PAGE. Electropherogram of proteins (0.3 μg) after purification (**b**).

Mutant forms of the *blp* gene were produced by site-directed mutagenesis. Expression plasmids carrying the genes for mutant forms of the Blp enzyme were electroporated into mutant strain *L. capsici* XL1Δ*blp*, yielding the expression strains *L. capsici* XL1Δ*blp* Blp, *L. capsici* Y160A, *L. capsici* Y160R, *L. capsici* R144A, *L. capsici* N136A, and *L. capsici* N136R (see Section 4.3, Section 4.4 and Section 4.5 of Materials and Methods). Proteins were purified from the culture fluid of the expression strains according to the developed scheme (Figure 3a) and in accordance with Materials and Methods. As a result, the bacteriolytic protease Blp and its mutant forms Y160A, Y160R, R144A, N136A, and N136R were obtained in a homogeneous form (Figure 3b).

**Figure 4:**
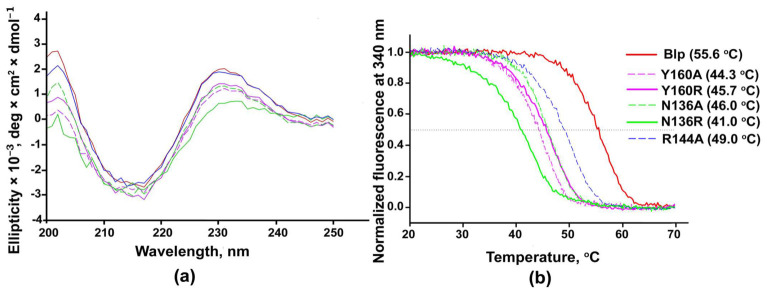
Secondary structure identity verification of the Blp protease and its mutant forms using CD and assessment of overall thermal stability using fluorescence. (**a**) Far-ultraviolet circular dichroism spectra of the bacteriolytic protease Blp and its mutant forms at 20 °C. All proteins were maintained under identical conditions in 30 mM Na-acetate buffer, pH 5.5, 0.3 M NaCl at a concentration of 0.7 mg mL^−1^. (**b**) Normalized dependences of the fluorescence intensity of protein solutions at 340 nm (excitation wavelength 280 nm) on the sample temperature. In brackets, melting temperatures of the enzymes.

Circular dichroism (CD) (Figure 4a) and fluorescence (Figure 4b) techniques were used to assess the effect of amino-acid point substitutions on the Blp structure.

Figure 4a shows the far-UV CD spectra. It can be seen that the CD spectra of all proteins are virtually identical. The shape of all spectra corresponds to beta-structural proteins. Calculation of the percentage content of elements of the secondary structure of the Blp protease and its mutant forms showed that the content of antiparallel beta sheets remains in a narrow range: 47.3% for wild-type Blp, 47.8% for R144A, 47.2% for N136A, 47.2% for Y160R, 45.3% for Y160A and 41.3% for N136R (Table S1). Thus, the mutations did not significantly affect the secondary structure of the native protein.

To determine the effect of mutations on the packing density of the Blp hydrophobic core, we studied protein melting using fluorescence. Fluorescence intensity vs. sample temperature curves are presented in Figure 4b. All melting curves are S-shaped, indicating cooperative protein melting, i.e., the presence of a dense hydrophobic core. Moreover, the melting temperatures of all mutant Blp forms decreased by 6.6–14.6 °C compared to the native Blp enzyme, which had a melting temperature of 55.6 °C (Figure 4b). This result indicates that the amino acid substitutions destabilized the Blp protease but did not disrupt the dense packing of the hydrophobic core.

Thus, the mutations did not affect the spatial structure of the protein but destabilized it.

### 2.3. Effect of Mutations on the Bacteriolytic and Proteolytic Activities of the Blp Protease

**Figure 5:**
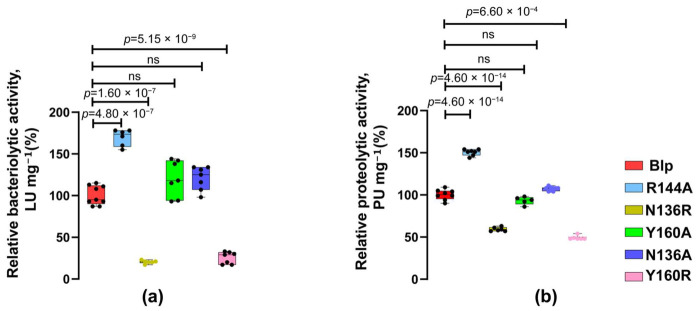
Effect of mutations on the bacteriolytic and proteolytic activities of the Blp protease. Comparative characteristics of the bacteriolytic (**a**) and proteolytic (**b**) activities of the native Blp protease and its mutant forms. Statistical analysis to compare bacteriolytic activity was performed using a Welch ANOVA followed by Tamhane’s T2 test for multiple comparisons, *p* = 5 × 10^−15^, W(5, 15.03) = 355.3. Data were considered significant at *p* < 0.05. Statistical analysis to compare the proteolytic activity of the Blp with R144A, N136A, N136R, and Y160A was performed using a one-way ANOVA with Tukey’s multiple comparison test, *p* < 1 × 10^−15^, F(4, 30) = 415. A two-sided Mann–Whitney U-test was used to compare the Blp and Y160R. Data were considered significant at *p* < 0.001. ns means not significant.

To measure bacteriolytic and proteolytic activities, living *S. aureus* 209P cells and casein, respectively, were used as substrates. As can be seen in Figure 5a,b, the substitution of tyrosine with arginine in the Y160R mutant protein led to a 4- and 2-fold decrease in bacteriolytic and proteolytic activities, respectively. In the N136R mutant protein, the substitution of asparagine with arginine led to a 5- and 2-fold decrease in these activities, respectively. In the Y160A and N136A mutant proteins, the substitution of tyrosine and asparagine with alanine did not affect the activity. The substitution of arginine with alanine in the R144A protein led to an increase in bacteriolytic and proteolytic activities by 1.7 and 1.5 times, respectively (Figure 5a,b).

### 2.4. Effect of Mutations on the Binding of the Blp to Living Cells and S. aureus 209P Peptidoglycan

**Figure 6:**
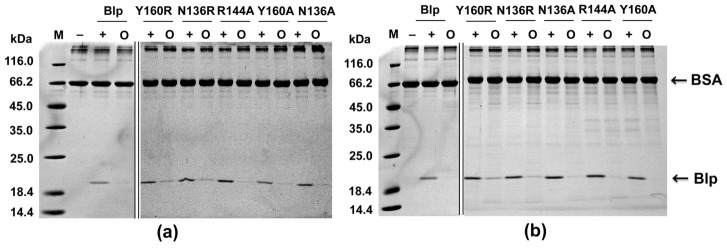
SDS-PAGE. Effect of mutations on the binding capacity of the Blp protease to substrate. Interaction of the bacteriolytic protease Blp and its mutant forms with *S. aureus* 209P peptidoglycan (**a**) and living *S. aureus* 209P cells (**b**). M, a mixture of protein standards; −, negative control (reaction mixture without the enzyme); +, positive control (reaction mixture without the substrate); O, enzyme–substrate interaction. An amount of 12 µL of sample was loaded into the gel. The major protein band above 116 kDa corresponds to the oligomeric form of BSA. The original gels are shown in Figures S2 and S3. The samples were derived from the same experiment; the gels were processed in parallel.

The effect of mutations was investigated using a method enabling the determination of the ability of mutant Blp forms to bind to living *S. aureus* 209P cells and its peptidoglycan (see Section 4.8 of Materials and Methods). It was found that substitutions of tyrosine with arginine in the Y160R mutant protein and asparagine with arginine in the N136R mutant protein led to a deterioration in their binding ability to *S. aureus* 209P peptidoglycan by 1.4 and 1.6 times, respectively (Figure 6a) and to living *S. aureus* 209P cells by 2.7 and 2.1 times, respectively (Figure 6b). In the Y160A, N136A, and R144A mutant proteins, substitutions of tyrosine and asparagine with alanine, as well as arginine with alanine, did not affect the binding capacity of the enzymes. The obtained data correlate with the results of measuring bacteriolytic and proteolytic activities described above (Figure 5a,b).

Thus, we identified in the Blp structure the amino acid residues of tyrosine at position 160 and asparagine at position 136 of the C-terminal subdomain, which can be responsible for the binding of this enzyme to the carbohydrate moiety of the peptidoglycan during the interaction of the enzyme with the surface of the target cell.

## 3. Discussion

In this work, we aimed to identify the region in the spatial structure of the Blp enzyme responsible for the initial interaction with the surface of target cells. This remains an unexplored issue for extracellular bacteriolytic proteases of Gram-negative bacteria. Currently, the best-studied enzymes in this regard are homologous enzymes of Gram-positive bacteria. The well-investigated lysostaphin from *S. simulans* biovar *staphylococcus* illustrates the modular organization of such enzymes. Lysostaphin has a substrate-binding C-domain (SH3b domain, prokaryotic sarcoma homology 3 binding domain), which is connected by a linker to the catalytic domain [10]. The SH3b domain has two binding sites, one for the pentaglycine bridge and the other for the peptide stem of the *S. aureus* peptide chain [11]. The known extracellular bacteriolytic proteases of Gram-negative bacteria do not have such substrate-binding domains, but it is obvious that the spatial structure of these proteins must contain sites of interaction with the target cell.

In 2020, the first study was published to identify the site of interaction between the bacteriolytic protease pseudoalterin from the Gram-negative bacterium *Pseudoalteromonas* sp. CF6-2 and target cell peptidoglycan [12]. The authors identified a segment in the region of amino acid residues N134–R173. In this segment, they established amino acid residues H133, N134, R141, N154, Y157, E159, R163, and R164, responsible for the enzyme’s binding to the carbohydrate moiety of target cell peptidoglycan. They also used a superposition of the pseudoalterin structure with the structures of lysostaphin and staphylolysin LasA for comparative analysis and concluded that pseudoalterin has a distinct substrate-binding C-domain. However, they mistakenly considered LasA to be an enzyme with a two-domain organization, which leads to the incorrect—in our opinion—conclusion about the presence of a C-domain in pseudoalterin. It is well known that the extracellular bacteriolytic protease LasA of the Gram-negative bacterium *Pseudomonas aeruginosa* has a single-domain organization, which contains a C-terminal subdomain [2]. We believe that it is more correct to compare pseudoalterin with LasA, since their primary sequences of mature parts are 53% identical, while with lysostaphin the identity is only 17%. The structural homology of pseudoalterin is significantly higher with LasA than with lysostaphin: the root mean square deviation index is 1.43 vs. 3.34, respectively. Thus, the amino acid residue region of N134–R173 for pseudoalterin identified by Tang and coauthors can be considered a C-terminal subdomain, rather than an independent C-domain, as described in the publication. Despite this, we believe their work to be pioneering and important in uncovering the mechanism of interaction between extracellular bacteriolytic proteases of Gram-negative bacteria and the surface of target cells.

In our study, we hypothesized that a similar structural and functional C-terminal subdomain may exist in the bacteriolytic protease Blp from *L. capsici* XL1, which we have studied. We should add here that we have previously discovered a structural homology between the bacteriolytic proteases Blp and LasA [7]. To search for such a region in the Blp, we performed a structural superposition of the Blp and pseudoalterin (Figure 1a). This revealed a region in the Blp structure bounded by residues N136–R167, structurally identical to the C-subdomain of pseudoalterin. We hypothesized that this C-terminal subdomain of the Blp could be responsible for the initial interaction of the enzyme with the carbohydrate moiety of target cell peptidoglycan, as in pseudoalterin.

MD simulations of the bacteriolytic protease Blp with the (NAG–NAM)_2_ ligand showed that the acetamide groups of the ligand were located predominantly in the pocket-like regions of the C-terminal subdomain, with lower hydrophilicity. This may be considered contradictory due to the generally hydrophilic nature of a substantial part of the amino acids found within the proposed peptidoglycan-binding region (e.g., asparagine, arginine, and threonine). However, the local surface properties affect the water free energy alongside the amino acids types. The numerous cavities that form the binding site trap water inside them, thus decreasing the solvent translational entropy and, as a consequence, the relative hydrophilicity of the corresponding region. The substitution of water molecules with acetamide groups is therefore driven by the entropic factor, which is part of the hydrophobic effect. Thus, the interaction of the C-terminal subdomain of the Blp with the ligand can be hydrophobic. Indeed, MD simulations revealed a relationship between the hydrophobicity/hydrophilicity profile of the C-terminal subdomain of mutant Blp forms upon interaction with the ligand. Point substitutions at positions R144, N136, and Y160 with alanine did not change the hydrophobicity/hydrophilicity profile of the C-terminal subdomains of the N136A and Y160A mutant proteins, while the area of hydrophobic regions in the R144A protein actually increased compared to the native Blp form. Point substitutions of amino acid residues with arginine in the N136R and Y160R mutant proteins led to a decrease in the hydrophobicity profile of the C-terminal subdomain and, accordingly, to a decrease in the area of the putative interaction of the Blp mutant forms with the ligand.

The data obtained using MD were confirmed experimentally. For this, we obtained mutant proteins with point substitutions of amino acid residues with alanine and arginine at positions Y160, R144, and N136 of the Blp’s C-terminal subdomain. Here, we should note an important stage of the work—the production of a homologous expression strain based on *L. capsici* XL1 cells with a deleted gene of the Blp enzyme for the subsequent expression of the mutant protein genes. In homologous expression systems for bacteriolytic enzymes of Gram-negative bacteria, it is possible to obtain “correctly folded” target proteins, since protein topogenesis occurs naturally in cells of the native producer [8,13,14]. The use of such an expression system was a prerequisite for a correct comparison of the mutant proteins. Thus, we obtained mutant proteins and conducted a comparative characterization of their binding capacity to peptidoglycan and living *S. aureus* 209P cells, as well as their specific activities. It turned out that the mutant proteins N136A, Y160A, and R144A, obtained by alanine mutagenesis, did not lose their ability to bind to peptidoglycan and living *S. aureus* 209P cells, and their antimicrobial and proteolytic properties were not impaired. However, the substitution of asparagine N136 and tyrosine Y160 with a more hydrophilic substituent, arginine, led to deterioration in the binding ability of the mutant forms of the Blp to peptidoglycan and living *S. aureus* 209P cells.

Thus, the results confirm the importance of hydrophobic interactions in the binding of the enzyme to peptidoglycan of the target cells. However, our data differ from those obtained for pseudoalterin. Firstly, in the case of pseudoalterin, all point substitutions of amino acids H133 (which corresponds to N136 in the Blp), N134 (G137), R141 (R144), N154 (S157), Y157 (Y160), E159 (T162), R163 (Q166), and R164 (N167) with alanine resulted in a decrease in the enzyme’s binding affinity. Secondly, Tang and coauthors used molecular docking to demonstrate that the interaction of the C-terminal subdomain of pseudoalterin with the (NAG–NAM)_2_ ligand occurs via hydrogen bonds.

Thus, we identified a C-terminal subdomain in the Blp structure, which can be involved in the initial binding of the enzyme to the peptidoglycan of target cells, likely through hydrophobic interactions. This subdomain contains amino acid residues N136 and Y160, which can be important in this interaction. However, more experimental data is needed for various bacteriolytic enzymes from Gram-negative bacteria to understand whether a common mechanism of interaction exists between these enzymes and target cells or whether these mechanisms are specific to individual enzymes.

## 4. Materials and Methods

### 4.1. Bacterial Strains and Cultivation Conditions

Cells of the *Lysobacter* strains were cultivated in liquid LB-M medium (g L^−1^): peptone, 5; yeast extract, 5; NaCl, 5; pH 7.5, at 29 °C for 21 h, with aeration at 205 rpm. LB-M agar medium contained 1.5% agar. When necessary, antibiotics Tc or Gm were added at a final concentration of 40 and 20 µg mL^−1^, respectively. Strain *Escherichia coli* DH5α was cultivated on LB medium containing 10 µg mL^−1^ Tc or 10 µg mL^−1^ Gm at 37 °C for 16–18 h. *S. aureus* 209P cells were cultivated on LB medium at 29 °C for 15–18 h.

### 4.2. Molecular Dynamics Simulation

The three-dimensional structure of the bacteriolytic protease Blp has been previously produced by us and deposited in the RCSB Protein Data Bank (ID 8AF1) [7]. Prior to MD simulations, crystallographic water and other associated molecules and ions were removed from the structure. To simulate the binding of the studied protein and its mutant forms to a fragment of the carbohydrate backbone of peptidoglycan (NAG–NAM)_2_ by MD, we constructed a general AMBER force field (GAFF2) set [15,16] for the NAM residue. The parameters of GLYCAM_06j [17] for the NAG residue were used as a basis, and the missing elements were obtained from the parameterization of the lactate anion within the GAFF2 force field [18] using the ACPYPE program (v: 2023.10.27) [19]. The final force field files for the whole oligosaccharide were generated in the tleap program, which is part of the AmberTools24 package [20], and then converted to the GROMACS format using the ParmEd Python library (v: 4.2.2) [21]. The preparation of the systems for molecular dynamics was performed on the CHARMM-GUI web server [22,23]. The protein or its complex with the (NAG–NAM)_2_ ligand was dissolved in a rectangular water cell, to which Na^+^ and Cl^−^ ions were also added at a concentration of 0.15 M. The AMBER19SB [24], GLYCAM_06j, and TIP3P [25,26] force fields were used to generate the topologies of the protein, sugars, and water, respectively.

All MD simulations were performed using the GROMACS 2024.3 software package [27]. A typical setup included using a V-rescale thermostat (T = 303.15 K, time constant 1 ps) and an isotropic C-rescale barostat (*p* = 1 bar, time constant 5 ps, compressibility 4.5 × 10^−5^ bar^−1^), constraining h-bonds with LINCS [28] and SETTLE [29] algorithms for solute and water, respectively, setting the non-bonded cutoff equal to 0.9 nm, dealing with long-range electrostatics with PME [30], and applying long-range dispersion correction for energy and pressure. The overall integration time step was 0.002 ps. Possible conformations of the protein–ligand complex were sampled using the simulated annealing method. In this case, we smoothly varied the oligosaccharide reference temperature from 303.15 to 350 K and back every 20–50 ns during a 200–500 ns simulation. The (NAG–NAM)_2_ ligand was held at a distance of no more than 0.15–0.2 nm from the binding site, for which appropriate constraints (upperWalls) were imposed on the distance between their centers of mass using the built-in Colvars module [31]. Calculation of trajectories for their subsequent analysis using the Grid Inhomogeneous Solvation Theory (GIST) method involved imposing positional constraints on the protein backbone atoms (force constant 40 kJ mol^−1^ nm^−2^).

The GIST methodology [32,33] was used to construct water free energy (WFE) surfaces around the Blp protease and its mutants. Long (500 ns, 50,000 frames) MD trajectories, pre-cleaned of ions and in which the positions of the protein backbone atoms were fixed and centered in the cell, were processed using the gist module of the cpptraj program (part of AmberTools24) [34]. The lattice volume and position were chosen to cover the entire protein and solvent at a distance of 2 nm, and the linear dimension of an elementary voxel was 0.05 nm. Analysis of output files was performed using the gisttools Python library (https://github.com/liedllab/gisttools) (accessed on 16 November 2024). The resulting water energy components (Eww, Esw, dTs_trans_, dTS_orient_) in each voxel were normalized to standard values for bulk TIP3P water. The free energy of water near each atom of the trajectory-averaged protein structure was calculated as a weighted average over all voxels within a 0.7 nm radius of the atom. The weight was determined by the distance to the atom and approximated by a Gaussian function (σ = 0.3 nm). Finally, the solvent-inaccessible surface (SES) of the protein was visualized in the VMD workspace (v. 1.9.4a57) [35] and colored depending on the previously obtained WFE values for each atom. The area accessible to the acetamide groups of the (NAG–NAM)_2_ ligand on the protein surface was defined as the fraction of the SES in the vicinity of 5 Å from which the groups were located for more than 90% of the MD time.

### 4.3. Kits, Strains and Plasmids for Molecular Genetic Manipulations

Use was made of Q5 DNA polymerase (New England Biolabs, Ipswich, MA, USA), T4 DNA ligase, T4 polynucleotide kinase, and restriction endonucleases *BamHI*, *HindIII*, *XhoI*, *SmaI*, and *XbaI* (Thermo Fisher Scientific, Waltham, MA, USA). We also used the QIAquick Gel Extraction Kit (Qiagen, Germantown, MD, USA), FastPure EndoFree Plasmid Mini Kit, and FastPure Gel DNA Extraction Mini Kit (Vazyme, Nanjing, China) to isolate DNA from agarose gels and bacterial cells.

Highly competent *E. coli* DH5α cells were obtained using the RbCl method [36]. Expression plasmids were transformed into competent *Lysobacter* cells by the previously optimized and modified Lin method [37] at 12.5 kV cm^−1^.

The oligonucleotides used in this work are listed in Table 1 and Table S2.

Oligonucleotides were designed using OligoAnalyzer (https://eu.idtdna.com/pages/tools/oligoanalyzer?returnurl=%2Fcalc%2Fanalyzer) (accessed on 20 February 2025) and synthesized at the Evrogen facility (Moscow, Russia).

The plasmids, as well as the expression and mutant strains, are presented in Table 2. The correct assembly of all constructs and the nucleotide sequences of the target genes were validated by sequencing at the Evrogen facility (Moscow, Russia).

### 4.4. Production of the Mutant Strain L. capsici XL1Δblp

All molecular genetic procedures were performed according to the kit manufacturers’ recommendations and the Sambrook and Russell manual [40].

Mutant strain *L. capsici* XL1Δ*blp* with a deletion in the *blp* gene was produced by homologous recombination according to our previously developed protocol [14,37]. Genomic DNA fragments of *L. capsici* XL1 containing the 5′ end of the *blp* gene with the flanking upstream region (1036 bp), as well as the 3′ end of the *blp* gene with the flanking downstream region (963 bp), were amplified using specific primers (Table S2). The upstream and downstream fragments were then sequentially cloned into the pJQ200SK suicide vector digested with *XhoI*/*SmaI* and *SmaI*/*XbaI* restriction endonucleases, respectively. Finally, the marked mutation of the Tc^R^ resistance cassette gene was introduced into the pJQ200SKΔ*blp* plasmid (Table 2) and digested with alkaline phosphatase and the *SmaI* restriction endonuclease. This resulted in the pJQ200SKΔ*blp::tet* plasmid (Table 2), which was electroporated into *L. capsici* XL1 cells. Clones with the Suc^S^Tc^R^Gm^R^ phenotype were selected after the first crossing over. The selected clones were then cultivated in LB-M medium at 29 °C to an optical density of 0.3 at 540 nm, followed by plating on LB-M medium containing 10% sucrose and 40 µg mL^−1^ Tc. Clones with the Suc^S^Tc^R^Gm^R^ phenotype, which indicated the secondary crossing over, were selected. Mutation was confirmed by the PCR using selective primers and sequencing (Table S2). The PCR of the selected clones and wild-type *L. capsici* XL1 DNA yielded amplicons of 2008 bp (Figure S4, lanes 1, 2) and 1663 bp (Figure S4, lane 4), respectively. This indicates a successful substitution of the *blp* gene segment between 39 and 1131 bp with the Tc^R^ cassette.

Characterization of strain *L. capsici* XL1Δ*blp* showed that deletion of the *blp* gene did not affect cell growth, but led to a decrease in the bacteriolytic activity of *L. capsici* XL1 against living target cells and a complete loss of lytic activity against living *S. aureus* 209P cells (Figures S5 and S6).

### 4.5. Production of the Mutant Forms of the Blp Gene

Mutant forms of the *blp* gene were produced using a site-directed mechanism in several stages according to Hilgarth and Lanigan [41], including an amplification of primary fragments (FR1 and FR2) (stage I), overlap PCR reaction (stage II), and final fusion-gene PCR amplification (stage III). To obtain each mutant form of the *blp* gene, two pairs of primers were used: internal primers with a single nucleotide substitution resulting in an amino acid substitution (Y160A, Y160R, R144A, N136A, and N136R) and flanking primers with recognition sites for the corresponding restriction endonucleases *HindIII* or *BamHI* (Table 1). Plasmid DNA pBBR1–MCS5 P_GroEL(A)_*–blp* [8] was used as a template for the PCR. The overlap PCR reaction step was performed using the following program: preliminary denaturation at 98 °C for 30 s, followed by 15 cycles of denaturation at 98 °C for 10 s, annealing of overlapping DNA fragments at 63 °C for 20 s, and elongation at 72 °C for 30 s. In the final, third step, the full-length gene of the mutant *blp* forms was obtained, which, after the treatment with restriction endonucleases *HindIII*/*BamHI*, was cloned into the pBBR1-MCS5 P_GroEL(A)_*–gfp* vector developed by us previously [8]. This resulted in expression plasmids that were electroporated into *L. capsici* XL1Δ*blp* cells. Validation of the expression plasmid assembly and the sequences of the gene of the mutant *blp* forms was carried out by sequencing.

### 4.6. Purification of the Bacteriolytic Protease Blp and Its Mutant Forms

Proteins were purified from the culture fluid of the expression strains *L. capsici* XL1Δ*blp* Blp, *L. capsici* Y160A, *L. capsici* Y160R, *L. capsici* R144A, *L. capsici* N136A, and *L. capsici* N136R after discarding the cells by centrifugation at 7000× *g* for 30 min. (NH_4_)_2_SO_4_ was added to 300 mL of the resulting culture fluid to 80% saturation at 4 °C and centrifuged at 22,470× *g* for 1 h. The resulting protein precipitate was suspended and dialyzed against 50 mM Tris-HCl, pH 8.0 for subsequent purification using column chromatography. For the first cation exchange chromatography, a Toyopearl CM-650 column (Merck, Darmstadt, Germany) equilibrated with 50 mM Tris-HCl, pH 8.0, was used. Proteins were eluted with 50 mM Tris-HCl, pH 8.0, containing 0.3 M NaCl. Fractions possessing bacteriolytic activity against living *S. aureus* 209P cells were pooled and dialyzed against 50 mM Tris-HCl, pH 8.0. In the second step, chromatography was performed on an ENrichS column (Bio-Rad, Hercules, CA, USA) connected to an NGC chromatography system (Bio-Rad, Hercules, CA, USA). Proteins were eluted with a linear NaCl gradient from 0.03 to 0.30 M. Protein fractions were analyzed by SDS-PAGE and bacteriolytic activity measurement. Lytically active fractions containing homogeneous enzymes were pooled, dialyzed against 30 mM Na-acetate buffer, pH 5.5, 0.3 M NaCl, and stored at −20 °C. Before studying the biochemical properties of the enzymes, the fractions were dialyzed against 10 mM Tris-HCl, pH 8.0. Enzyme concentrations were determined by the Bradford method [42] using the calibration curve of absorbance at 595 nm vs. concentration, constructed for BSA (Merck, Darmstadt, Germany) in the range of 1–25 µg mL^−1^, using an iMark immunoassay analyzer (Bio-Rad, Hercules, CA, USA).

To study bacteriolytic and proteolytic activities, as well as binding abilities, the concentration of all enzymes was adjusted to 0.05 mg mL^−1^; to determine CD spectra, to 0.7 mg mL^−1^.

### 4.7. Circular Dichroism and Fluorescence Measurements

CD measurements were performed on a J-1500 spectrometer (JASCO, Tokyo, Japan). All spectra were measured using 0.1 mm path-length cuvettes at the protein concentration of 0.7 mg mL^−1^. Calculation of the percentage content of elements of the secondary structure of the Blp protease and its mutant forms was performed in the BestSel program (https://bestsel.elte.hu/index.php) (accessed on 20 December 2025) [43,44].

The protein fluorescence measurements were performed using a Cary 100 spectrometer (Varian, Palo-Alto, CA, USA) with standard 1 cm path-length quartz cuvettes, 2 mL sample volume. The excitation wavelength was 280 nm. The melting curves were recorded at 325, 340, 360 nm at the protein concentration of 0.1 mg mL^−1^.

### 4.8. Determination of the Binding Capacity of Enzymes with Peptide Glycoprotein and S. aureus Cells

The binding capacity of the Blp protease and its mutant forms to substrates was assessed using the method described by Sekiya et al. [45], with a modification. The substrates were *S. aureus* 209P peptidoglycan, which we previously obtained using the method of Shaw et al. [46] with a modification, and living *S. aureus* 209P cells. The 30-μL reaction mixture contained: substrate (14 μL peptidoglycan at OD_540_ = 0.5, or 6 μL living cells at OD_540_ = 50), 10 μL native Blp enzyme or its mutant forms (0.5 μg), and 2 μL BSA (4 μg). The reaction mixture was prepared on ice and immediately centrifuged at 17,000× *g* for 3 min at 4 °C to prevent rapid hydrolysis of the substrates and to detect only the binding event. Then, 12 μL of the supernatant was collected and analyzed electrophoretically.

Each reaction was performed in at least three biological replicates.

### 4.9. Determination of Bacteriolytic Activity

Bacteriolytic activity was determined by turbidimetry. Living *S. aureus* 209P cells grown as described in Section 4.1 were used as a substrate. After cultivation, the cells were washed with 10 mM Tris-HCl, pH 8.0 by centrifugation at 7000× *g*, and the cell suspension density was brought to OD_540_ = 0.5 with the same buffer. The reaction mixture was prepared on ice: native Blp protease or its mutant forms (0.01–0.09 μg) were added to 1 mL of the substrate. As a control, the appropriate amount of 10 mM Tris-HCl, pH 8.0, buffer was added to the substrate. The mixture was then incubated at 37 °C for 5 min, after which the tubes were placed back on ice. Measurements were performed at 540 nm using a UV-160A spectrophotometer (Shimadzu, Kyoto, Japan).

Bacteriolytic activity (LU mg^−1^) was calculated using the formula:

[0.5 (OD_540_ of the control suspension) − OD_540_ of the experimental suspension] × 1000 × V (total reaction volume) × dilution/[min (reaction time) × V (enzyme volume) × 0.01 (correction coefficient for the OD reduction per min) × mg mL^−1^ of enzyme].

All measurements were performed in three biological replicates, each with at least two technical replicates.

### 4.10. Determination of Proteolytic Activity

Proteolytic activity was determined according to the Hull method [47].

200 µL of the Blp protease or its mutant forms (2.1–5.7 µg) was added to 200 µL of 1% casein (Merck, Darmstadt, Germany) in 10 mM Tris-HCl, pH 8.0, and the mixture was incubated at 37 °C for 5 min. Then, 800 µL of 5% TCA was added to the reaction mixture to inactivate the enzymes. A control sample was added with 200 µL of the enzyme sample after 800 µL of 5% TCA. All samples were again incubated at 37 °C for 10 min and centrifuged at 17,000× *g* for 10 min, after which the absorbance was measured at 280 nm.

Proteolytic activity (PU mg^−1^) was measured using the following formula:

[µmol (tyrosine equivalents released) × 1.2 mL (total reaction volume) × n (dilution of enzyme samples)]/[5 min (reaction time) × 0.2 mL (sample volume) × mg mL^−1^ of enzyme].

The concentration dependence curve of a tyrosine solution (Merck, Darmstadt, Germany) at 280 nm was used as a calibration curve.

Measurements were performed in three biological replicates, each with at least two technical replicates.

### 4.11. Electrophoresis

#### 4.11.1. SDS-PAGE

Protein electrophoresis was performed in 12.5% PAG in the presence of SDS according to the Laemmli method [48]. A mixture of SM0431 protein standards (Thermo Fisher Scientific, Waltham, MA, USA) was used as a marker: β-galactosidase, 116.0 kDa; BSA, 66.2 kDa; ovalbumin, 45.0 kDa; lactate dehydrogenase, 35.0 kDa; REase Bsp981, 25.0 kDa; β-lactoglobulin, 18.4 kDa; lysozyme, 14.4 kDa. Protein bands were stained in the gel with 0.05% Coomassie Brilliant Blue R-250 (Merck, Darmstadt, Germany).

The analysis of the color intensity of protein bands was performed using the GelAnalyzer 23.1.1 program (http://www.gelanalyzer.com/?i=1) (accessed on 10 October 2025) in at least three biological replicates.

#### 4.11.2. DNA Electrophoresis in 0.8% Agarose Gel

Separation of PCR products was performed in 0.8% agarose gel in 1× TAE buffer containing 0.5 mg mL^−1^ ethidium bromide at 100 V. GeneRuler Mix SM0331 (Thermo Fisher Scientific, Waltham, MA, USA) was used to determine the length of the separated DNA fragments.

### 4.12. Statistical Analysis

Statistical analysis was performed using GraphPad Prism version 8.0.1 (GraphPad Software, San Diego, CA, USA). Data are presented as means ± standard deviations in the form of boxplots (medians ± interquartile spans). For the normally distributed data of two groups, the two-sided unpaired Student’s *t*-test was used; for other data types, the two-sided Mann–Whitney *U*-test. To compare more than two groups with a normal distribution and equal variances, we used a one-sided analysis of variance (ANOVA), followed by the Tukey test for multiple comparison; for unequal variances, the Welch’s ANOVA test with the Tamhane T2 test for multiple comparison was used.

## Figures and Tables

**Table 1:** Oligonucleotides used to produce mutant forms of the *blp* gene.

Mutation	Oligonucleotides	Amplification of *blp* Gene Fragments
Y160A	GGATCCTCAGTTCGGGCCTGGG GCCGGTTCGCGCTGACCAAGAACGGTCA	FR1, 71 bp
	AAGCTTATGAAGGCGATTTCGGGAGC GTCAGCGCGAACCGGCTGCAGTTGG	FR2, 1082 bp
Y160R	GGATCCTCAGTTCGGGCCTGGG AGCCGGTTCCGCCTGACCAAGAACGGTCA	FR1, 72 bp
	AAGCTTATGAAGGCGATTTCGGGAGC GTCAGGCGGAACCGGCTGCAGTTGG	FR2, 1082 bp
R144A	GGATCCTCAGTTCGGGCCTGGG GTCGGGCTATGCGATCACCGCGACCGG	FR1, 121 bp
	AAGCTTATGAAGGCGATTTCGGGAGC CGGTGATCGCATAGCCCGACAGGTAGGTGC	FR2, 1036 bp
N136A	GGATCCTCAGTTCGGGCCTGGG TACCACCTCGCGGGCACCTACCTGT	FR1, 144 bp
	AAGCTTATGAAGGCGATTTCGGGAGC GTGCCCGCGAGGTGGTAGAAGCTGCCG	FR2, 1010 bp
N136R	GGATCCTCAGTTCGGGCCTGGG TACCACCTCCGCGGCACCTACCTGT	FR1, 144 bp
	AAGCTTATGAAGGCGATTTCGGGAGC GTGCCGCGGAGGTGGTAGAAGCTGCCG	FR2, 1010 bp
	GGATCCTCAGTTCGGGCCTGGG AAGCTTATGAAGGCGATTTCGGGAGC	Full-length mutant *blp* gene, 1137 bp

**Table 2:** Strains and plasmids used.

Plasmids	Characteristics	References
pJQ200SK	Suicide vector with the *sacB* gene, GmR	[38]
pBR322	Source of Tc^R^	[39]
pBBR1–MCS5 P_GroEL(A)_–*gfp*	Strain pBBR1-MCS5 with the *gfp* gene under the control of the modified promoter GroEL *L. enzymogenes*	[8]
pBBR1–MCS5 P_GroEL(A)_–*blp*	Plasmid pBBR1-MCS5 with the *blp* gene under the control of the modified promoter GroEL *L. enzymogenes*	[8]
pBBR1–MCS5 P_GroEL(A)_–*blpY160A*, pBBR1–MCS5 P_GroEL(A)_–*blpY160R*, pBBR1–MCS5 P_GroEL(A)_–*blpR144A*, pBBR1–MCS5 P_GroEL(A)_–*blpN136A*, pBBR1–MCS5 P_GroEL(A)_–*blpN136R*	Plasmid pBBR1-MCS5 with the mutant *blp* gene under the control of the modified promoter GroEL *L. enzymogenes*	This work
pJQ200SKΔ3′*blp*	pJQ200SK with a 5′ fragment of the *blp* gene and the upstream flanking region	This work
pJQ200SKΔ*blp*	pJQ200SK with a 1091 bp deletion in the *blp* gene	This work
pJQ200SKΔ*blp::tet*	pJQ200SK with a deletion in the *blp* gene, marked with the Tc^R^ resistance cassette	This work
*L. capsici* XL1Δ*blp*	Strain *L. capsici* XL1 with a 1091 bp deletion in the *blp* gene	This work
*L. capsici* XL1Δ*blp* Blp	Strain *L. capsici* XL1 with a deletion in the *blp* gene, containing the plasmid pBBR1–MCS5 P_GroEL(A)_–*blp*	This work
*L. capsici* Y160A *L. capsici* Y160R *L. capsici* R144A *L. capsici* N136A *L. capsici* N136R	Strains *L. capsici* XL1 with a deletion in the *blp* gene, containing the plasmids pBBR1–MCS5 P_GroEL(A)_–*blpY160A*; pBBR1–MCS5 P_GroEL(A)_–*blpY160R*; pBBR1–MCS5 P_GroEL(A)_–*blpR144A*; pBBR1–MCS5 P_GroEL(A)_–*blpN136A*; pBBR1–MCS5 P_GroEL(A)_–*blpN136R*, respectively	This work
*E. coli* DH5α	F−, *φ* 80d*lacZ*∆M15, ∆(*lacZYA-argF*)U169, *deoR*, *recA1*, *endA1*, *hsdR17*(rK−, mK+), *phoA*, *supE44*, *λ−*, *thi-1*, *gyrA96*, *relA1*	[36]

## Data Availability

The original contributions presented in this study are included in the article/Supplementary Materials. Further inquiries can be directed to the corresponding author.

## Supplementary Materials

The following supporting information can be downloaded at: https://www.mdpi.com/article/10.3390/ijms27125246/s1.

## References

[1] Hedstrom L. (2002). Serine protease mechanism and specificity. Chem. Rev..

[2] Spencer J., Murphy L.M., Conners R., Sessions R.B., Gamblin S.J. (2010). Crystal structure of the LasA virulence factor from *Pseudomonas aeruginosa*: Substrate specificity and mechanism of M23 metallopeptidases. J. Mol. Biol..

[3] Rawlings N.D., Barrett A.J., Thomas P.D., Huang X., Bateman A., Finn R.D. (2018). The MEROPS database of proteolytic enzymes, their substrates and inhibitors in 2017 and a comparison with peptidases in the PANTHER database. Nucleic Acids Res..

[4] Whitaker D.R., Roy C., Tsai C.S., Jurásek L. (1965). Lytic enzymes of *Sorangium* sp. A comparison of the proteolytic properties of the alpha- and beta-lytic proteases. Can. J. Biochem..

[5] Afoshin A.S., Kudryakova I.V., Borovikova A.O., Suzina N.E., Toropygin I.Y., Shishkova N.A., Vasilyeva N. (2020). Lytic potential of *Lysobacter capsici* VKM B-2533T: Bacteriolytic enzymes and outer membrane vesicles. Sci. Rep..

[6] Afoshin A.S., Konstantinov M.A., Toropygin I.Y., Kudryakova I.V., Vasilyeva N.V. (2020). β-lytic protease of *Lysobacter capsici* VKM B-2533T. Antibiotics.

[7] Afoshin A., Tishchenko S., Gabdulkhakov A., Kudryakova I., Galemina I., Zelenov D., Leontyevskaya E., Saharova S., Leontyevskaya N. (2022). Structural and functional characterization of β-lytic protease from *Lysobacter capsici* VKM B-2533T. Int. J. Mol. Sci..

[8] Kudryakova I., Afoshin A., Leontyevskaya E., Leontyevskaya N. (2022). The first homologous expression system for the β-lytic protease of *Lysobacter capsici* VKM B-2533T, a promising antimicrobial agent. Int. J. Mol. Sci..

[9] Razew A., Schwarz J.N., Mitkowski P., Sabala I., Kaus-Drobek M. (2022). One fold, many functions-M23 family of peptidoglycan hydrolases. Front. Microbiol..

[10] Sabala I., Jagielska E., Bardelang P.T., Czapinska H., Dahms S.O., Sharpe J.A., James R., Than M.E., Thomas N.R., Bochtler M. (2014). Crystal structure of the antimicrobial peptidase lysostaphin from *Staphylococcus simulans*. FEBS J..

[11] Gonzalez-Delgado L.S., Walters-Morgan H., Salamaga B., Robertson A.J., Hounslow A.M., Jagielska E., Sabała I., Williamson M.P., Lovering A.L., Mesnage S. (2020). Two-site recognition of *Staphylococcus aureus* peptidoglycan by lysostaphin SH3b. Nat. Chem. Biol..

[12] Tang B.L., Yang J., Chen X.L., Wang P., Zhao H.L., Su H.N., Li C.Y., Yu Y., Zhong S., Wang L. (2020). A predator-prey interaction between a marine *Pseudoalteromonas* sp. and Gram-positive bacteria. Nat. Commun..

[13] Yu Z.C., Tang B.L., Zhao D.L., Pang X., Qin Q.L., Zhou B.C., Zhang X.Y., Chen X.L., Zhang Y.Z. (2015). Development of a cold-adapted *Pseudoalteromonas* expression system for the *Pseudoalteromonas* proteins intractable for the *Escherichia coli* system. PLoS ONE.

[14] Kudryakova I., Afoshin A., Leontyevskaya E., Leontyevskaya N. (2025). Development of efficient expression systems for bacteriolytic proteases L1 and L5 of *Lysobacter capsici* XL1. Int. J. Mol. Sci..

[15] Hornak V., Abel R., Okur A., Strockbine B., Roitberg A., Simmerling C. (2006). Comparison of multiple Amber force fields and development of improved protein backbone parameters. Proteins.

[16] Wang X., Xiong D., Zhang Y., Zhai J., Gu Y.C., He X. (2025). The evolution of the Amber additive protein force field: History, current status, and future. J. Chem. Phys..

[17] Bernardi A., Faller R., Reith D., Kirschner K. (2019). ACPYPE update for nonuniform 1–4 scale factors: Conversion of the GLYCAM06 force field from AMBER to GROMACS. SoftwareX.

[18] Wang J., Wolf R.M., Caldwell J.W., Kollman P.A., Case D.A. (2004). Development and testing of a general amber force field. J. Comput. Chem..

[19] Sousa da Silva A.W., Vranken W.F. (2012). ACPYPE—AnteChamber PYthon Parser interfacE. BMC Res. Notes.

[20] Case D.A., Aktulga H.M., Belfon K., Cerutti D.S., Cisneros G.A., Cruzeiro V.W.D., Forouzesh N., Giese T.J., Götz A.W., Gohlke H. (2023). AmberTools. J. Chem. Inf. Model..

[21] Shirts M.R., Klein C., Swails J.M., Yin J., Gilson M.K., Mobley D.L., Case D.A., Zhong E.D. (2017). Lessons learned from comparing molecular dynamics engines on the SAMPL5 dataset. J. Comput. Aided Mol. Des..

[22] Jo S., Kim T., Iyer V.G., Im W. (2008). CHARMM-GUI: A web-based graphical user interface for CHARMM. J. Comput. Chem..

[23] Lee J., Hitzenberger M., Rieger M., Kern N.R., Zacharias M., Im W. (2020). CHARMM-GUI supports the Amber force fields. J. Chem. Phys..

[24] Tian C., Kasavajhala K., Belfon K.A.A., Raguette L., Huang H., Migues A.N., Bickel J., Wang Y., Pincay J., Wu Q. (2020). ff19SB: Amino-acid-specific protein backbone parameters trained against quantum mechanics energy surfaces in solution. J. Chem. Theory Comput..

[25] Jorgensen W.L., Chandrasekhar J., Madura J.D., Impey R.W., Klein M.L. (1983). Comparison of simple potential functions for simulating liquid water. J. Chem. Phys..

[26] Chen A.A., Pappu R.V. (2007). Parameters of monovalent ions in the AMBER-99 forcefield: Assessment of inaccuracies and proposed improvements. J. Phys. Chem. B.

[27] Abraham M.J., Murtola T., Schulz R., Pall S., Smith J.C., Hess B., Lindahl E. (2015). GROMACS: High performance molecular simulations through multi-level parallelism from laptops to supercomputers. SoftwareX.

[28] Hess B. (2008). P-LINCS: A parallel linear constraint solver for molecular simulation. J. Chem. Theory Comput..

[29] Miyamoto S., Kollman P.A. (1992). Settle: An analytical version of the SHAKE and RATTLE algorithm for rigid water models. J. Comput. Chem..

[30] Essmann U., Perera L., Berkowitz M.L., Darden T., Lee H., Pedersen L.G. (1995). A smooth particle mesh Ewald method. J. Chem. Phys..

[31] Fiorin G., Klein M.L., Hénin J. (2013). Using collective variables to drive molecular dynamics simulations. Mol. Phys..

[32] Nguyen C.N., Young T.K., Gilson M.K. (2012). Grid inhomogeneous solvation theory: Hydration structure and thermodynamics of the miniature receptor cucurbit[7]uril. J. Chem. Phys..

[33] Chen L., Cruz A., Roe D.R., Simmonett A.C., Wickstrom L., Deng N., Kurtzman T. (2021). Thermodynamic decomposition of solvation free energies with particle mesh ewald and long-range lennard-jones interactions in grid inhomogeneous solvation theory. J. Chem. Theory Comput..

[34] Roe D.R., Cheatham T.E. (2013). PTRAJ and CPPTRAJ: Software for processing and analysis of molecular dynamics trajectory data. J. Chem. Theory Comput..

[35] Humphrey W., Dalke A., Schulten K. (1996). VMD: Visual molecular dynamics. J. Mol. Graph..

[36] Hanahan D. (1983). Studies on transformation of *Escherichia coli* with plasmids. J. Mol. Biol..

[37] Kudryakova I.V., Afoshin A.S., Ivashina T.V., Suzina N.E., Leontyevskaya E.A., Leontyevskaya N. (2021). Deletion of alpB gene influences outer membrane vesicles biogenesis of *Lysobacter* sp. XL1. Front. Microbiol..

[38] Quandt J., Hynes M.F. (1993). Versatile suicide vectors which allow direct selection for gene replacement in gram-negative bacteria. Gene.

[39] Balbás P., Soberón X., Merino E., Zurita M., Lomeli H., Valle F., Flores N., Bolivar F. (1986). Plasmid vector pBR322 and its special-purpose derivatives—A review. Gene.

[40] Sambrook J., Russell D.W. (2001). Molecular Cloning: A Laboratory Manual.

[41] Hilgarth R.S., Lanigan T.M. (2019). Optimization of overlap extension PCR for efficient transgene construction. MethodsX.

[42] Bradford M.M. (1976). A rapid and sensitive method for the quantitation of microgram quantities of protein utilizing the principle of protein–dye binding. Anal. Biochem..

[43] Micsonai A., Wien F., Murvai N., Nyiri M.P., Balatoni B., Lee Y.H., Molnár T., Goto Y., Jamme F., Kardos J. (2025). BeStSel: Analysis site for protein CD spectra-2025 update. Nucleic Acids Res..

[44] Kardos J., Nyiri M.P., Moussong É., Wien F., Molnár T., Murvai N., Tóth V., Vadászi H., Kun J., Jamme F. (2025). Guide to the structural characterization of protein aggregates and amyloid fibrils by CD spectroscopy. Protein Sci. A Publ. Protein Soc..

[45] Sekiya H., Okada M., Tamai E., Shimamoto T., Shimamoto T., Nariya H. (2021). A putative amidase endolysin encoded by *Clostridium perfringens* St13 exhibits specific lytic activity and synergizes with the muramidase endolysin Psm. Antibiotics.

[46] Shaw D.R., Mirelman D., Chatterjee A.N., Park J.T. (1970). Ribitol teichoic acid synthesis in bacteriophage-resistant mutants of *Staphylococcus aureus* H. J. Biol. Chem..

[47] Hull M.E. (1947). Studies on milk proteins. II. Colorimetric determination of the partial hydrolysis of the proteins in milk. J. Dairy Sci..

[48] Laemmli U.K. (1970). Cleavage of structural proteins during the assembly of the head of bacteriophage T4. Nature.

