# Supplementary material for: Peculiarities of the Interaction of the Bacteriolytic Protease Blp from Lysobacter capsici XL1 with the Cell Wall of Staphylococcus aureus 209P
Source: Int J Mol Sci. 2026 Jun 10;27(12):5246. doi: 10.3390/ijms27125246 (PMC13300490; doi:10.3390/ijms27125246)
Supplement: Supplementary file 1 [file ijms-27-05246-s001.zip › ijms-4317852-SI.pdf]

Peculiarities of the interaction of the bacteriolytic protease Blp from *Lysobacter capsici* XL1 with the cell wall of *Staphylococcus aureus* 209P

Irina Kudryakova [1], Alexey Afoshin [1], Egor Bulavko [2], Dmitry Ivankov [2], Bogdan Melnik [3], Elena Leontyevskaya [1] and Natalia Leontyevskaya [1,*]

1 Laboratory of Microbial Cell Surface Biochemistry, G.K. Skryabin Institute of Biochemistry and Physiology of Microorganisms, FRC PSCBR, Russian Academy of Sciences, 5 Prosp. Nauki, 142290 Pushchino, Russia; kudryakovairina@yandex.ru (I.K.); alex080686@mail.ru (A.A.); ealeont@gmail.com (E.L.)
2 Center for Bio- and Medical Technologies, Moscow, 121205, Russia; e.bulavko@ligandpro.ru (E.B.); ivankov13@gmail.com (D.I.)
3 Institute of Protein Research, Russian Academy of Sciences, 4 Institutskaya Str., Pushchino, Moscow Region, 142290, Russia; bmelnik@phys.protres.ru (B.M.)
* Correspondence: vasilyevanv@rambler.ru (N.L.)

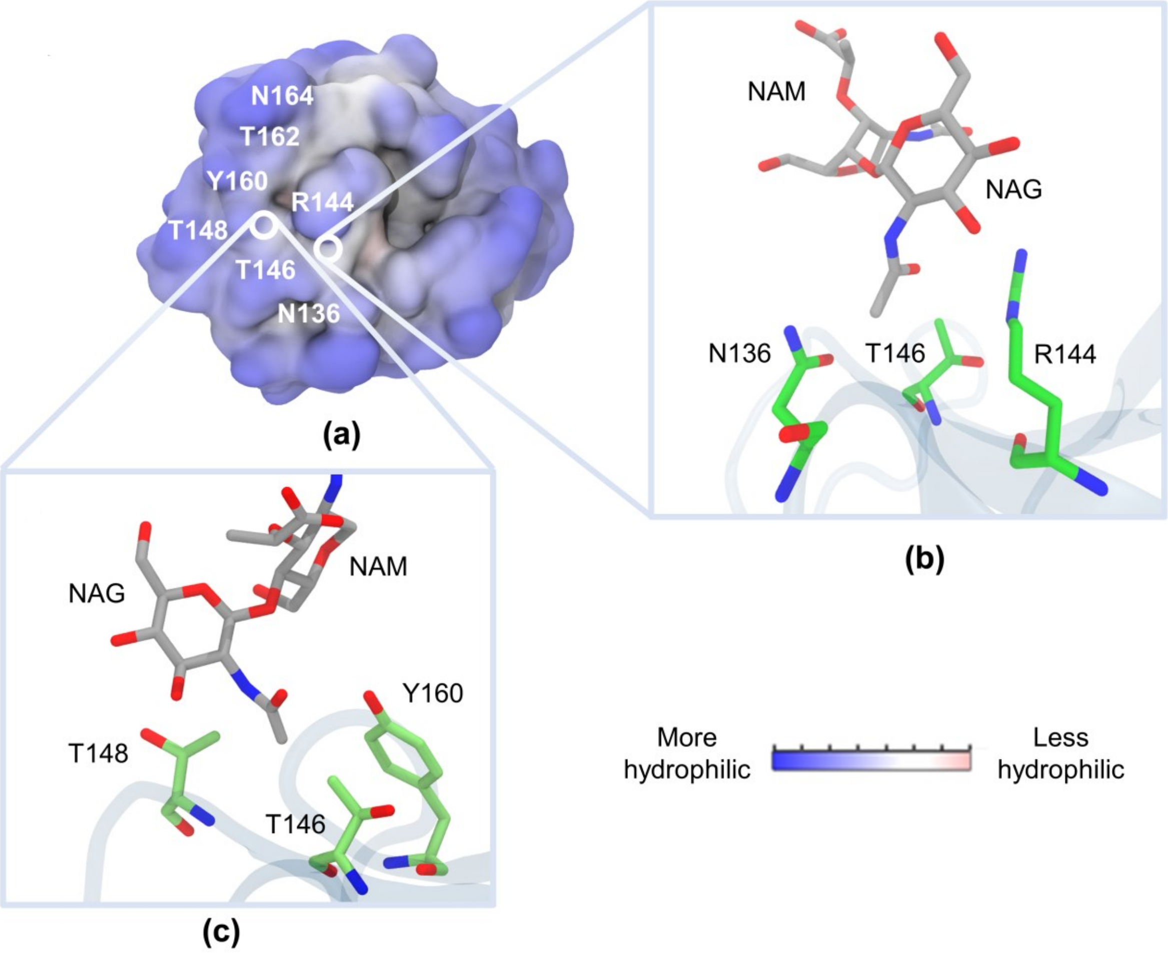

Figure S1. Superficial and structural organization of peptidoglycan binding site within C-terminal subdomain of the bacteriolytic protease Blp. Hydrophobicity/hydrophilicity map **(a)** indicates the location of less hydrophilic pocket-like structures, which interact with acetamide groups of $(NAG–NAM)_2$ ligand (**b, c**).

Table S1. Content of the secondary structure elements of the Blp protease and its mutant forms (%).

Bacteriolytic enzymes	Helix	Antiparallel	Parallel	Turn	Other
Blp	0.6	47.3	0	10.9	41.2
Y160A	1.1	45.3	0	11.6	42
Y160R	0.2	47.2	0	10.9	41.7
N136A	0.5	47.2	0	11.8	40.5
N136R	2	41.3	0	13.3	43.4
R144A	0	47.8	0	11.4	40.8

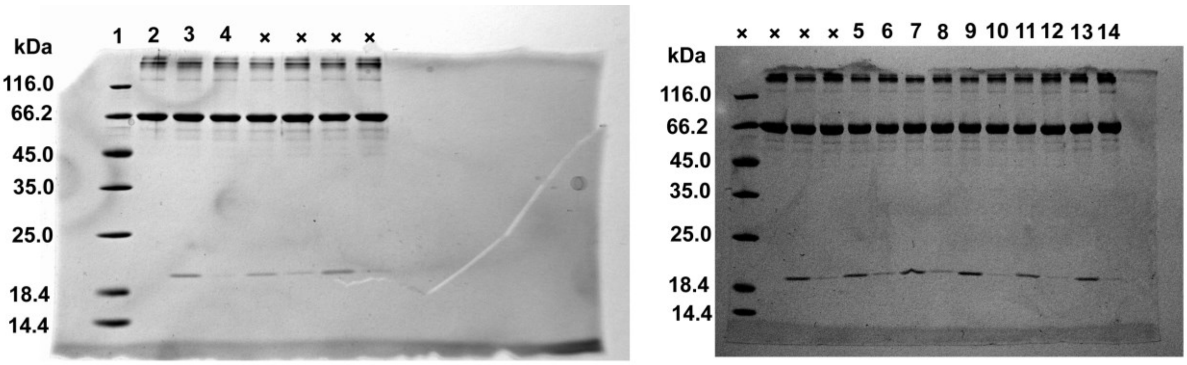

Figure S2. Original gel images for Figure 6a. Lane 1 corresponds to M of Figure 6a; lane 2, to the negative control of Figure 6a; lanes 3 and 4, to the reaction mixture with the Blp of Figure 6a; lanes 5 and 6, to the reaction mixture with Y160R of Figure 6a; lanes 7 and 8, to the reaction mixture with N136R of Figure 6a; lanes 9 and 10, to the reaction mixture with R144A of Figure 6a; lanes 11 and 12, to the reaction mixture with Y160A of Figure 6a; lanes 13 and 14, to the reaction mixture with N136A of Figure 6a.

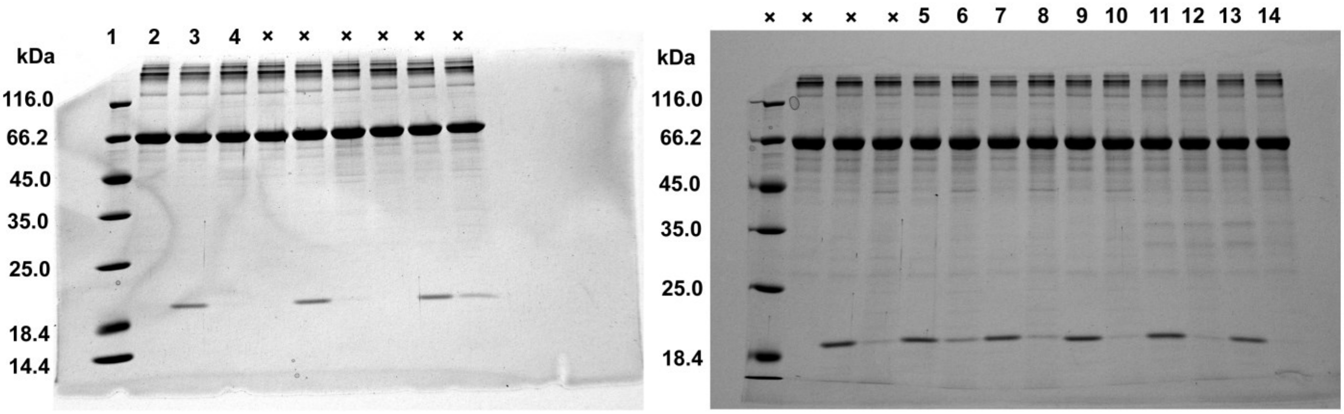

Figure S3. Original gel images for Figure 6b. Lane 1 corresponds to M of Figure 6b; lane 2, to the negative control of Figure 6b; lanes 3 and 4, to the reaction mixture with the Blp of Figure 6b; lanes 5 and 6, to the reaction mixture with Y160R of Figure 6b; lanes 7 and 8, to the reaction mixture with N136R of Figure 6b; lanes 9 and 10, to the reaction mixture with N136A of Figure 6b; lanes 11 and 12, to the reaction mixture with R144A of Figure 6b; lanes 13 and 14, to the reaction mixture with Y160A of Figure 6b.

Table S2. Oligonucleotides used in the work for knockout of the *blp* gene.

Oligonucleotides	Purpose
CTCGAGCCGATCCGCGTTG<br>CCCGGGCCAACAGCGTGATCCT	Amplification of a 1036-bp fragment<br>(5′ end of the *blp* gene and its flanking upstream region).
CCCGGGAACTGAGGTCGGCGCG<br>TCTAGACATGGAAACGGGCGTG	Amplification of a 963-bp fragment<br>(3′ end of the *blp* gene and its flanking upstream region).
GAATTCTCATGTTTGACAGCTTATCATCGA<br>CCCGAGATGCGCCG	Amplification of a 1433-bp Tc cassette from plasmid pBR322.
GCGAGGGGCGGATTATGC<br>CAGCTCCGATGCGATGACG	Validation of a deletion in the *blp* gene, marked with a 1430-bp Tc^R cassette.

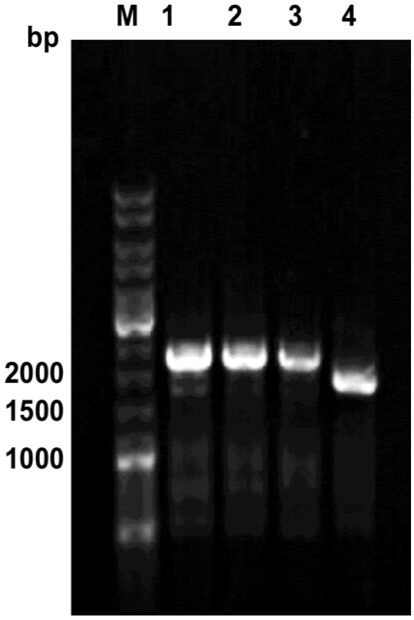

Figure S4. Electropherogram of PCR products. As a template, use was made of selected clones with the Suc^{R}Tc^{R}Gm^S phenotype (lanes 1, 2), plasmid DNA pJQ200SKΔ*blp::tet* (lane 3), and DNA of *L. capsici* XL1 (lane 4). M, markers.

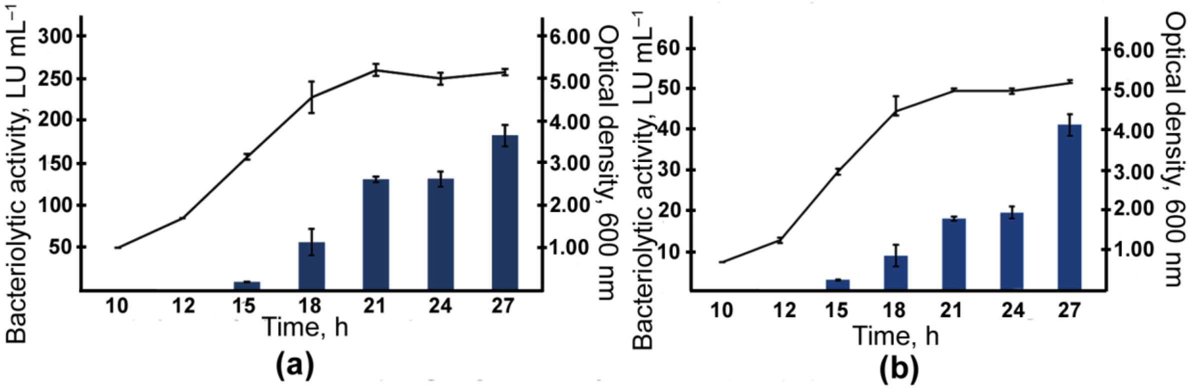

Figure S5. Dynamics of growth and bacteriolytic activity of wild-type strain *L. capsici* XL1 **(a)** and mutant strain *L. capsici* XL1Δ*blp* **(b)** in relation to autoclaved cells of *Staphylococcus aureus* 209P.

The growth dynamics and development of the bacteriolytic activity of mutant strain *L. capsici* XL1Δ*blp* and wild-type strain *L. capsici* XL1 did not differ (Figure S5). However, the total bacteriolytic activity in the culture fluid of the mutant strain was 7 times lower than that of the wild-type strain. The bacteriolytic activity against living cells of *Micrococcus luteus* Ac-2230^T and *Kocuria rosea* Ac-2200^T also decreased by 9 and 4 times, respectively (Figure S6). Mutant strain *L. capsici* XL1Δ*blp* completely lost bacteriolytic activity against living cells of *S. aureus* 209P.

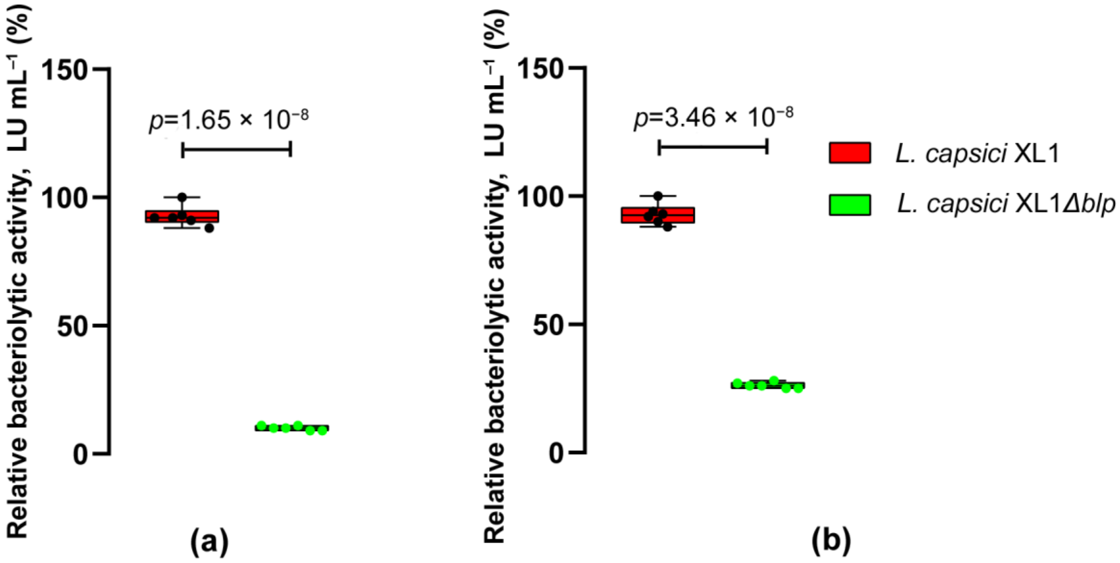

Figure S6. Comparative bacteriolytic activity of the culture fluid of *L. capsici* XL1 and *L. capsici* XL1Δ*blp* strains against living cells of *Micrococcus luteus* Ac-2230^{T} **(a)** and *Kocuria rosea* Ac-2200^{T} **(b).**
The mean values were obtained from two biological replicates, each performed in three technical replicates. Statistical analysis was performed using an unpaired two-tailed Student's *t*-test with Welch's correction: $t = 38.14$, $df = 5.8$ **(a);** $t = 49.60$, $df = 5.5$ **(b).** Data were considered statistically significant at $p < 0.05$.
